# Ataxin-2 as a candidate blood biomarker for estimating disease status in cases of suspected glioblastoma recurrence

**DOI:** 10.1007/s10014-025-00517-z

**Published:** 2025-09-22

**Authors:** Farida Garaeva, Riho Nakajima, Sho Tamai, Kensuke Tateishi, Akitake Mukasa, Shinji Kawabata, Hiroaki Nagashima, Manabu Natsumeda, Nozomi Hirai, Shota Tanaka, Shigeo Ohba, Nayuta Higa, Yoshiki Arakawa, Akihide Kondo, Hidehiro Kohzuki, Shinichiro Koizumi, Yutaka Fujioka, Tatsuya Abe, Hemragul Sabit, Masashi Kinoshita, Yasuo Uchida, Sumio Ohtsuki, Mitsutoshi Nakada

**Affiliations:** 1 Department of Neurosurgery, Graduate School of Medical Science, Kanazawa University, 13-1 Takara-Machi, Kanazawa, 920-8641 Japan; 2 Department of Genetics, Institute of Fundamental Medicine and Biology, Kazan Federal University, Kazan, Russia; 3 Department of Occupational Therapy, Faculty of Health Science, Institute of Medical, Pharmaceutical and Health Sciences, Kanazawa University, Kanazawa, Japan; 4 Department of Neurosurgery, Graduate School of Medicine, Yokohama City University, Yokohama, Japan; 5 Department of Neurosurgery, Graduate School of Medical Sciences, Kumamoto University, Kumamoto, Japan; 6 Department of Neurosurgery, Osaka Medical and Pharmaceutical University, Osaka, Japan; 7 Department of Neurosurgery, Kobe University Graduate School of Medicine, Kobe, Japan; 8 Department of Neurosurgery, Brain Research Institute, Niigata University, Niigata, Japan; 9 Department of Neurosurgery, Toho University Ohashi Medical Center, Tokyo, Japan; 10 Department of Neurosurgery, Graduate School of Medicine, The University of Tokyo, Tokyo, Japan; 11 Department of Neurological Surgery, Okayama University Graduate School of Medicine, Dentistry, and Pharmaceutical Sciences, Okayama, Japan; 12 Department of Neurosurgery, Fujita Health University School of Medicine, Toyoake, Japan; 13 Department of Neurosurgery, Graduate School of Medical and Dental Sciences, Kagoshima University, Kagoshima, Japan; 14 Department of Neurosurgery, Kyoto University Graduate School of Medicine, Kyoto, Japan; 15 Department of Neurosurgery, Faculty of Medicine, Juntendo University, Tokyo, Japan; 16 Department of Neurosurgery, University of Tsukuba Hospital, Tsukuba, Japan; 17 Department of Neurosurgery, Hamamatsu University School of Medicine, Hamamatsu, Shizuoka Japan; 18 Department of Neurosurgery, Graduate School of Medical Sciences, Kyushu University, Fukuoka, Japan; 19 Department of Neurosurgery, Saga University, Saga, Japan; 20 Department of Molecular Systems Pharmaceutics, Graduate School of Biomedical and Health Sciences, Hiroshima University, Hiroshima, Japan; 21 Department of Pharmaceutical Microbiology, Faculty of Life Sciences, Kumamoto University, Kumamoto, Japan

**Keywords:** Glioblastoma, Recurrence, Pseudoprogression, Biomarker, Ataxin-2

## Abstract

Differentiating pseudoprogression (PsP) from recurrence in cases of glioblastoma (GBM) after chemoradiotherapy is challenging, with neuroimaging as the only non-invasive method. In this study, we aimed to identify a blood biomarker for precise disease monitoring and investigated the role of Ataxin-2 (ATXN2). Blood samples (n = 45) from patients with suspected recurrence, including eight with PSP, were analyzed. In addition, tumor tissue samples (n = 22), including those from seven patients who also provided blood samples, were examined. Protein levels were assessed using quantitative proteomics and ELISA. ATXN2 levels were measured via western blotting, and localization was determined through immunohistochemistry and immunocytochemistry. ATXN2 knockdown was performed in glioma cell lines to assess its effects on proliferation, migration, and invasion. Proteomics identified ATXN2 as a potential biomarker. ELISA showed significantly higher serum ATXN2 levels in recurrence than in PsP (p = 0.028). ATXN2 ≥ 11.0 ng/mL and ≥ 8 months post-chemoradiotherapy distinguished recurrence from PsP (AUC = 0.82, sensitivity = 67.6%, specificity = 87.5%). ATXN2 was highly expressed in GBM tissues, localized in neurons and glioma cells, and its knockdown enhanced proliferation, migration, and invasion via ERK phosphorylation. ATXN2, highly expressed in GBM, may serve as a potential blood biomarker for distinguishing PsP from recurrence.

**Supplementary Information:**

The online version contains supplementary material available at 10.1007/s10014-025-00517-z.

## Introduction

Glioblastoma (GBM) is the most aggressive form of brain tumor, with a prognosis and a median overall survival of < 15 months despite surgery, chemotherapy with temozolomide, and radiotherapy [1, 2]. Such aggressive treatment may lead to post-therapeutic tissue changes characterized by necrosis and inflammation, termed pseudoprogression (PsP), which can be detected using gadolinium-enhanced lesions during magnetic resonance imaging (MRI) [3–6]. However, determining whether the gadolinium-enhanced lesions represent PsP or recurrence is challenging in clinical practice.

Currently, several MRI-based methods are used in clinical practice to diagnose recurrence, often involving a combination of MRI sequences such as diffusion-weighted imaging (DWI), perfusion MRI, and magnetic resonance spectroscopy [7, 8]. Various other methods have been applied, but their clinical utility remains limited given restrictions in the national insurance system or the low number of hospitals with imaging equipment. For example, amide proton transfer MRI is a promising diagnostic method with high accuracy [5]. Positron emission tomography (PET) imaging, including methionine-PET and fludeoxyglucose-18 (^18^F) PET, is considered effective for differentiating between recurrence and PsP [9]. Amino-acid PET tracers such as ^11^C-MET also exhibit high sensitivity for differentiating tumor recurrence from treatment-related changes (i.e., PsP) in high-grade gliomas [10].

Several attempts have been made to identify circulating biomarkers for GBM [11]. For instance, cell-free DNA and microRNA are circulating biomarkers whose levels correlate with tumor burden and can help distinguish between recurrence and treatment-related changes [12]. However, no reliable liquid biomarker has been discovered to date because despite their potential, the use of cell-free nucleic acids as biomarkers is limited, owing to their typical low concentrations in body fluids, complicating their detection. In GBM, tumoral components (proteins, cell-free nucleic acids, and circulating tumor cells) are released into the blood and cerebrospinal fluid [13, 14]. Therefore, detecting these substances in the blood of a patient could aid algorithms in distinguishing GBM recurrence from PsP.

In this context, we hypothesize that blood biomarkers could offer a promising diagnostic approach with the advantages of rapid detection and ease of use in clinical practice. Ataxin-2 (ATXN2) is widely expressed in the central nervous system [15]. Mutation of the polyglutamine tract in the *ATXN2* gene causes spinocerebellar ataxia type 2 and is one of the risk factors for various other neurodegenerative diseases, including amyotrophic lateral sclerosis and Parkinson’s disease [16]. Its role in brain tumors, however, remains poorly understood. Therefore, with a focus on the ATXN2 protein as a translational molecule, we aimed to identify a candidate molecule as a potential blood biomarker that can distinguish between PsP and recurrence. The rationale was to examine ATXN2 through the sequential window acquisition of all theoretical fragment ion spectra (SWATH) modes as a liquid biomarker for estimating disease status.

## Materials and methods

### MR images and definition of diagnosis

MR images, including cadmium-enhanced T1-weighted, DWI, and arterial spin labeling (ASL) images, were acquired as a part of standard care, before and immediately after surgery, and every three months. These images were acquired using conventional, high-resolution, three-dimensional sequences on a 3.0-T MRI scanner (Signa Excite HDx 3.0 T [GE Healthcare, Little Chalfont, UK] or Philips Ingenia [Philips Healthcare, Best, the Netherlands]). According to the RANO 2.0 criteria [17], patients exhibiting progression of an enhanced lesion in the first 12 weeks after completion of radiotherapy, provided it develops within the radiation field, had a high probability of PsP. However, some studies suggest that PsP can occur during the first 6 months after chemo-radiotherapy [3], extending beyond 12 weeks. In this case of suspected PsP, we performed advanced imaging techniques such as DWI, ASL, and PET imaging. However, a PET study is not covered by national insurance in Japan, and hence, some patients refused to undergo PET because of its cost. To prevent misclassification in this study, the final decision of true progression and PsP was determined through a retrospective review of clinical course, and PsP was diagnosed only upon confirmation of reduction or disappearance of enhanced region during observation periods without any additional treatment (except for temozolomide maintenance therapy for standard care).

### Serum and tumor tissue samples

Pooled serum samples collected from the patients with GBM at recurrence (n = 11) and PsP (n = 3) diagnosis. The samples were used for proteomic analysis using data-independent acquisition (DIA) to identify potential biomarkers. The median age of the patients was 66.4 ± 8.6 years. Serum samples for enzyme-linked immunosorbent assay (ELISA) were obtained from 45 patients with GBM (age: 61.2 ± 12.7 years) at the point of appearance of the gadolinium-enhanced lesion after chemo-radiotherapy using MRI. Blood samples were collected in EDTA tubes and stored on ice until further processing. Within 30 min after collection, the samples were centrifuged at 4 °C, 3000 rpm for 5 min, and the supernatant was stored at − 30 °C.

Brain tumor tissues with CNS WHO grade 2 (n = 8), grade 3 (n = 8), and grade 4 (GBM, n = 22) and non-neoplastic brain tissue adjacent to tumors (n = 10) were obtained during neurosurgical resection. Samples were frozen at − 80 °C before use. Tissue samples used for paraffin embedding were immersed in 4% paraformaldehyde. Tumors were classified according to WHO criteria 2021 [1].

### Quantitative proteomic analysis

Each sample was measured with a single replicate. Plasma samples were diluted fivefold with 0.1 M Tris–HCl (pH 8.5), and 5 mL of diluted plasma was added to 15 mL of 8 M urea in 0.1 M Tris–HCl (pH 8.5). The proteins were reduced with DTT and alkylated with iodoacetamide. Then, the sample was diluted to 1.2 M urea with 0.1 M Tris–HCl (pH 8.5). The protein was first digested with 0.5 mg lysyl endopeptidase (Fujifilm Wako) for 3 h at 30 °C and subsequently digested with 0.5 mg trypsin for 16 h at 37 °C. The digested peptide was desalted using self-packed StageTips with a CDS Empore SDB-XD sheet (GL Science, Tokyo, Japan), and the peptides were finally eluted with 0.5% formic acid in 50% acetonitrile. After drying, the peptides were reconstituted with 0.1% formic acid in 2% acetonitrile, and peptide concentration was measured using a BCA assay (Thermo Fisher Scientific, Waltham, MA, USA). A digested peptide sample (1 µg peptide) was injected into liquid chromatography–tandem mass spectrometry (MS).

The data acquired using data-dependent acquisition (DDA) for protein identification and spectral library generation and DIA/SWATH modes for protein quantification on a TripleTOF6600 (SCIEX, Framingham, MA, USA) coincided with those obtained using the Eksigent NanoLC400 system (SCIEX). The injected sample was loaded on a trap column (L-column ODS, 5 mm length × 0.3 mm ID, CERI, Tokyo, Japan) and separated in a self-packed emitter column (150 mm length × 75 mm ID) packed with ReproSil C18 (3 μm, Dr. Maisch, Ammerbuch, Germany). The mobile phases comprised (A) 0.1% formic acid in water and (B) 0.1% formic acid in acetonitrile. A linear gradient of 2–35% B for 120 min was employed for both DDA and DIA. For DDA, precursor ions were scanned from 400 to 1250 with an accumulation time of 250 ms, and product ions were scanned from 100 to 1600 with an accumulation time of 50 ms using rolling collision energy. The maximum number of candidate precursor ions for monitoring product ions was 30 ions/cycle, and analyzed ions were excluded for 15 s. For DIA, precursor ions were scanned from 400 to 1250 with an accumulation time of 50 ms, and product ions were scanned from 100 to 1600 with 100 variable isolation windows with rolling collision energy. The cycle time was 3.10 s.

Proteins were identified using ProteinPilot v.5 (SCIEX) with MS data from DDA and UniProt Human reference proteome data with default settings. The data identified from ProteinPilot were imported into PeakView (SCIEX), and a spectral library was generated to analyze the DIA data. DIA-NN version 1.8 was used to analyze the peaks of the peptides with DIA data [18] with default settings using a spectral library constructed as described above. Identified proteins and precursors were filtered by less than 1% FDR. Cross-run normalization was performed using the RT-dependent method built into DIA-NN.

### Article selection and literature review

Two authors (S.T. and R.N.) conducted a systematic literature search for articles on the relationship between glioma and the candidate proteins in the PubMed database. Information was extracted from the titles and abstracts to determine whether the articles reported on the relationship between the candidate proteins and glioma. Candidate proteins were listed in an Excel file for further analysis. The review was performed according to Preferred Reporting Items for Systematic Reviews and Meta-Analyses (PRISMA).

### ELISA

Serum ATXN2 levels were quantified using competitive ELISA (Human Ataxin 2 ELISA Kit; MyBioSource, San Diego, CA, USA). Serum samples were diluted (1:10) in phosphate-buffered saline (PBS) (pH 7.0–7.2), and 100 µL/well of the samples and appropriate standards were added to a 96-well plate. An equal volume of PBS was used as the blank control. A conjugate solution (50 µL) was added to each well (excluding the blank control). The plates were then incubated for 1 h at 37 °C. Thereafter, the plate was washed five times with a wash solution (350 µL/well/wash). Subsequently, the liquid from each well was removed, and 50 µL of substrates A and B were added to each well, including the blank control. The plate was covered and incubated for 15 min at 37 °C. Next, 50 µL of the stop solution was added to each well, including the blank control. The absorbance was immediately determined at 450 nm using a microplate reader. The absolute concentration of the protein was calculated from the standard curve.

### Western blot analysis

The tissue samples were lysed in a radioimmunoprecipitation assay buffer (Fujifilm, Osaka, Japan) containing protease and phosphatase inhibitors (Sigma-Aldrich, St. Louis, MO, USA) and incubated at 4 °C for 20 min. Following sonication on ice twice, samples were centrifuged for 10 min at 14,000 rpm and 4 °C. The protein concentration was calculated using a bicinchoninic acid protein assay kit (Thermo Fisher Scientific). Western blot analysis was performed as previously described [19]. Proteins from GBM cell lines were extracted using the same procedures. The following antibodies were used: anti-ATXN2 (GTX130329, 1:3000; Gene Tex, Irvine, CA, USA), anti-p-ERK (4370S, 1:3000; Cell Signaling Technology, Danvers, MA, USA), anti-ERK (4695S, 1:3000; Cell Signaling Technology), anti-AKT (9272S, 1:1000; Cell Signaling Technology), anti-p-AKT (4058S, 1:1000; Cell Signaling Technology), anti-mTOR (2983S, 1:1000; Cell Signaling Technology), anti-p-mTOR (2971S, 1:1000; Cell Signaling Technology), anti-STAT3 (12640S, 1:1000; Cell Signaling Technology), and anti-p-STAT3 (9134S, 1:1000; Cell Signaling Technology). β-actin was used as a loading control and for calculating the relative density values.

### Cell culture

Three human GBM cell lines—U87, U251, and T98G—were used. The cells were purchased from the European Collection of Authenticated Cell Cultures. The cells were maintained in Dulbecco’s modified Eagle’s medium (DMEM) supplemented with 10% heat-inactivated fetal bovine serum (FBS) at 37 °C in an incubator comprising 5% CO_2_.

### Immunohistochemistry and immunocytochemistry

For immunohistochemistry, 4-µm-thick, paraffin-embedded surgical specimen slices were deparaffinized and autoclaved in target retrieval solution (pH 6.0) at 120 °C for 10 min, treated with 3% hydrogen peroxide in methanol for 30 min, and blocked with 5% skimmed milk for 1 h. The slides were incubated with primary antibodies (1:300 dilution, anti-ATXN2 GTX130329; GeneTex) and immunostained using an Envision + kit (K4003, Dako, Agilent Technologies, Santa Clara, CA, USA). The stained sections were visualized using a 3,3ʹ-diaminobenzidine tetrahydrochloride solution for 5 min.

For immunocytochemistry, cells seeded onto circular glass coverslips with a 24-mm diameter at 80% confluence were fixed in 4% paraformaldehyde (168–20,955, Wako Pure Chemicals, Osaka, Japan) for 20 min at 4 °C, washed with PBS twice, and blocked in 5% skimmed milk for 60 min at room temperature (18–25 °C). The cells were treated with anti-ATXN2 (GTX130329; GeneTex) diluted 1:150 in a pre-dilution solution (S3022, Dako, Agilent Technologies) as the primary antibody and covered with a coverslip and incubated in a humidified chamber overnight. The slides were then washed with PBS and incubated for 60 min in a humidified chamber at room temperature with the Alexa Fluor 546-conjugated secondary antibody (1:1000 dilution, A-11035, Thermo Fisher Scientific). The cells were counterstained with a DAPI-containing mounting medium (UltraCruz^®^ Aqueous Mounting Medium with DAPI: sc-24941, Dallas, TX, USA).

### *ATXN2* gene silencing using small interfering RNA (siRNA)

To silence the target proteins, we used two different sequences of siRNAs targeting *ATXN2* and a negative control (Qiagen, Germantown, MD, USA). The target sequence 1 of human *ATXN2* (siATXN2-5, SI04192566, Qiagen) was 5ʹ-AAGACGCAGCTGAGCAAGTTA-3ʹ, and the target sequence 2 of human ATXN2 (siATXN2-6, S04292841, Qiagen) was 5ʹ-AGAGGTCGAAACAGTAACAAA-3ʹ. All cells were seeded in flat-bottomed culture plates at a density of 1.5 × 10^5^ cells per well in 2 mL of 10% FBS P/S. After 24 h, the medium was replaced with an all-free medium. U87, U251, and T98G cell lines were transfected with *ATXN2*-targeting and control siRNAs using Avalanche Omni (EZ Biosystems, College Park, MD, USA), following the manufacturer’s protocol. The final siRNA concentration was 25 nM. Cells were incubated for 48 h after transfection and used for subsequent experiments.

### Cell proliferation assay

To assess the proliferation of GBM cells (1 × 10^3^) treated with siRNA, the cells were passaged into a 96-well plate with 200 µL of culture medium containing 0.5% FBS. After incubating at 37 °C for 4 h, 20 µL of Alamar blue (Biosource, Camarillo, CA, USA) was added to each well. The plates were read using a microplate reader at 0, 24, 48, 72, and 96 h. The average fluorescence values from six wells were calculated and plotted.

### Cell migration and invasion assays

To investigate the effect of ATXN2 on glioma cell motility, we performed a migration assay using an uncoated Transwell chamber (6.5 mm diameter, 8 μm pore size). The chambers were pre-incubated in DMEM at 37 °C for 2 h. Glioma cells (1.0 × 10^5^ cells) were added to the upper chamber containing FBS-free DMEM. The Transwell chamber containing cells was incubated in DMEM with 10% FBS for 6 h. The cells that migrated to the lower surface of the chamber were fixed with methanol and stained using the Diff-Quik kit (Sysmex, Co. Ltd., Kobe, Japan). The numbers of invading and migrating cells in five microscopic fields were randomly selected and counted.

The invasion activity was assessed using a Matrigel pre-coated invasion Transwell chamber (6.5 mm diameter, 8 μm pore size; Corning, Bedford, MA, USA). After 2 h of pre-incubation in DMEM at 37 °C, 1.5 × 10^5^ GBM cells were resuspended in DMEM without FBS, and penicillin–streptomycin was added to each well. The Transwell chamber was placed in a 24-well plate (Corning), and DMEM containing 10% FBS was added. After 12 h of incubation, non-invasive cells on the upper surface of the filter were removed, and invasive cells on the lower surface were fixed with methanol and stained using a Diff-Quik kit.

### Statistical analysis

Parametric and paired t-tests were used to compare serum ATXN2 levels between the groups. The patients with recurrence were classified into two groups based on the lower and higher expression. Statistical significance for migration, invasion, and proliferation assays was calculated using Dunnett’s tests. We used non-parametric methods since the data did not follow a normal distribution. Multiple logistic regression analyses were performed and receiver operating characteristic (ROC) curves were constructed to investigate the association between serum ATXN2 and disease status. All statistical analyses were performed using GraphPad Prism software version 9.1.2 (MDF Co., Tokyo, Japan) and the JMP Pro statistical analysis software Pro version 16.2.0 (SAS Institute Japan Inc., Tokyo, Japan).

## Results

### Identification of marker candidates for distinguishing PsP from recurrence

To identify plasma marker candidates for distinguishing PsP, we analyzed plasma samples from 3 and 11 patients with PsP and recurrence. Demographic and clinical factors in these patients are summarized in Supplementary Table 1.

**Fig. 1:**
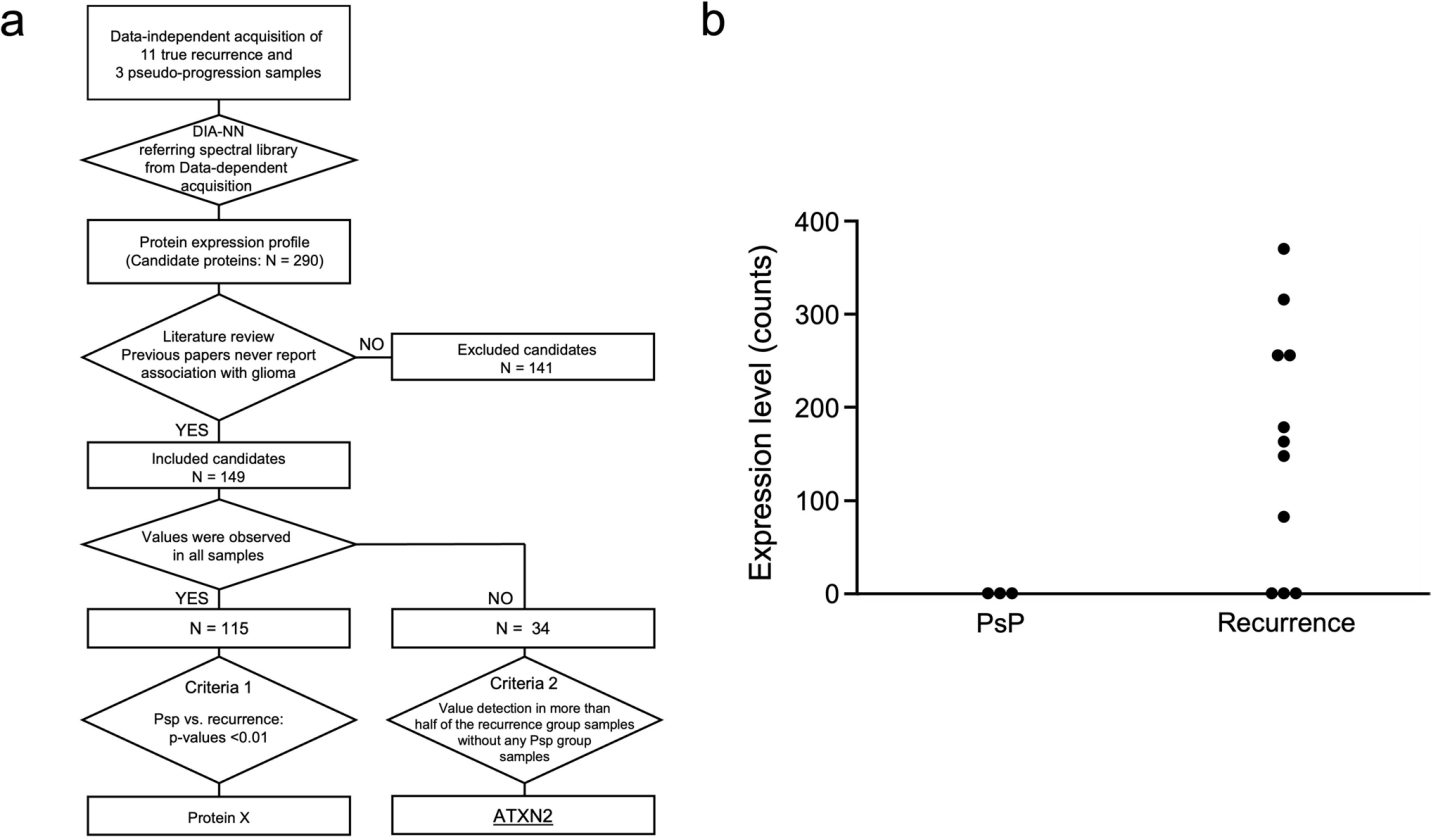
ATXN2 was identified as a biomarker candidate using sequential window acquisition of all theoretical fragment ion spectra (SWATH) mass spectrometry. **a.** Flow chart showing method for identifying ATXN2. Desalted plasma samples were analyzed using liquid chromatography–tandem mass spectrometry in data-dependent acquisition and data-independent acquisition (DIA)/SWATH modes. Based on mass spectrometry data, 290 proteins were identified, and SWATH data were analyzed with a spectral library constructed from identification data. Following literature review, 149 proteins were included as candidates without any association with glioma reported. The candidates were divided into two groups focusing on the value of the samples. Protein X and ATXN2 were selected as biomarker candidates. **b.** ATXN2 was highly expressed in serum samples of patients with GBM recurrence, whereas its levels were below the detection threshold in patients with pseudoprogression (PsP). Each sample had one replicate for measurement. DIA-NN, data-independent acquisition neural networks

An overview of our procedure for identifying candidate proteins is provided in Fig. 1a. The spectral library, which was generated from DDA, was used to analyze the DIA data, and we identified 290 candidate proteins (Supplementary Table 2). For the literature review, 11,971 papers were identified for the 290 candidate proteins (Supplementary Table 3). After screening the content of the studies for eligibility, 149 candidate proteins without any publications examining the relationship between them and glioma were included (Supplementary Table 3). We then classified the 149 candidate proteins into two groups depending on whether quantitative values were observed in all samples because some proteins were not detected due to their values being below the detection limit, which made statistical analysis of those proteins difficult. Consequently, 115 proteins were detected in all samples, whereas 34 proteins were not consistently detected.

For proteins detected in all samples, we selected candidate proteins based on two criteria: (1) proteins with a p-value of < 1% when comparing the PsP and recurrence groups using a t test (Supplementary Table 4) [20, 21]. As a result, we identified one protein, X. The average level of protein X in the recurrence group was 1.78-fold higher than that in the PsP group, representing a significant difference (p = 0.0084, Supplementary Fig. 1). Although some previous reports have reported the expression of protein X in glioma cells, none have identified it as a biomarker, and a parallel validation study is currently ongoing. Therefore, the name of the protein has been withheld to maintain confidentiality and will be disclosed in a subsequent publication. (2) Proteins were selected if they were not detected (below the detection limit) in any samples of the PsP group but were detected in more than half of the recurrence group samples (Supplementary Table 3) [22, 23]. Using this criterion, we identified another protein, ATXN2, which was detected in 8 of the 11 recurrence samples but in none of the PsP samples (Fig. 1b). To support these results, dynamic proteomic profiling revealed that the serum levels of ATXN2 were elevated at diagnosis, decreased significantly following initial treatment, and increased again at the time of true tumor recurrence (Supplementary Fig. 2). In contrast, ATXN2 levels remained low during episodes of PsP, indicating a potential role in distinguishing true progression from treatment-related changes. Therefore, in this study, we focused on investigating the utility of ATXN2 as a blood biomarker.

### *Validation of ATXN2 expression *via* ELISA*

**Fig. 2:**
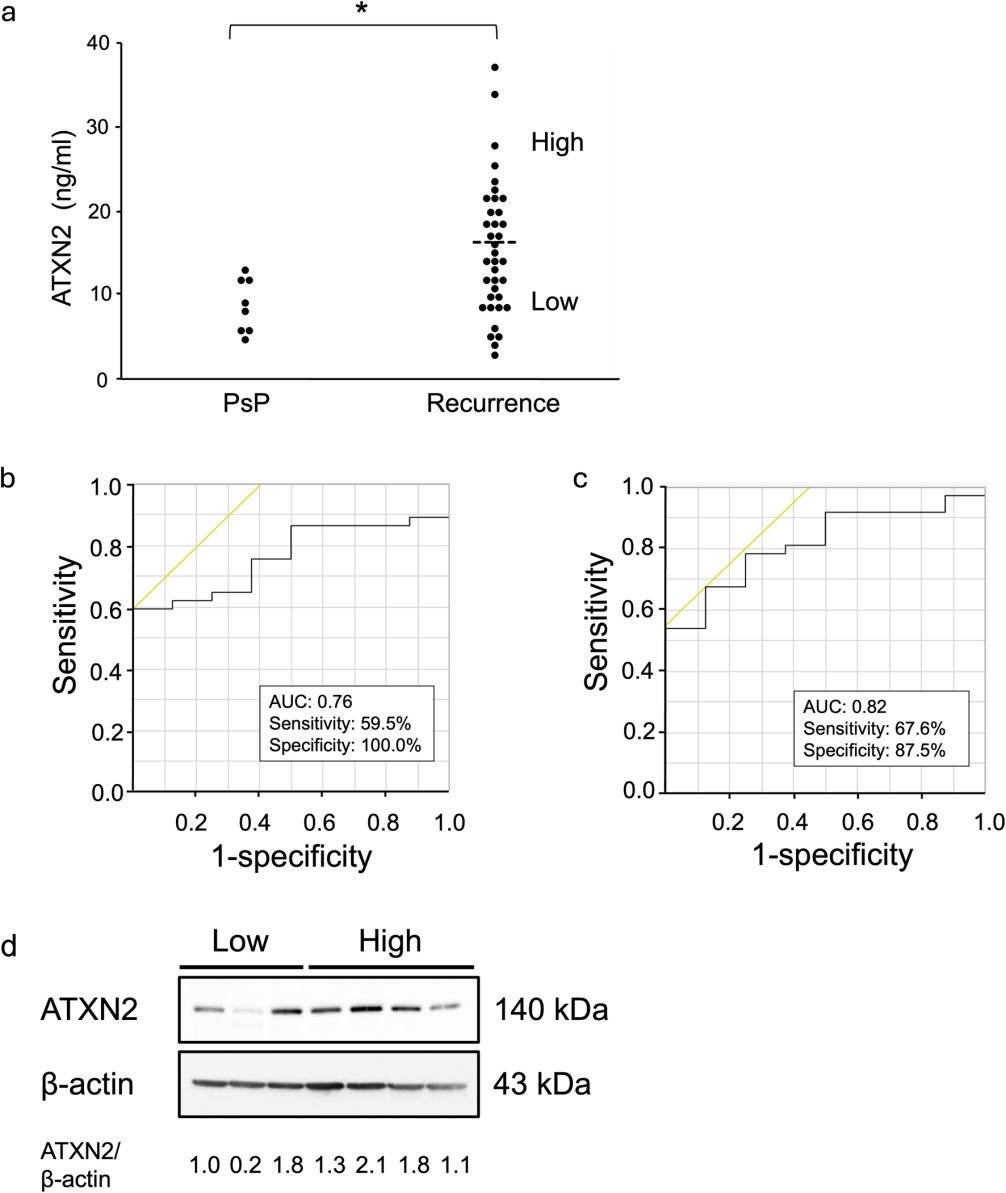
ATXN2 expression in plasma and GBM tissues. **a**. ATXN2 is highly expressed in patients with recurrence compared to those with pseudoprogression (PsP). Recurrence samples were divided into groups with higher and lower expression based on the concentration threshold (dotted line). *p < 0.05. **b.** Receiver operating characteristic (ROC) analyses were performed using ATXN2 expression as an explanatory variable. **c.** ATXN2 expression and months after the chemoradiotherapy. **d.** ATXN2 expression detected using western blot analysis in tissue samples from the low-expression group (n = 3) and high-expression group (n = 4). ATXN2 signal intensity (indicated values) was normalized to that of β-actin in the same lane (ATXN2/β-actin). AUC, Area under the curve

The marker performance of ATXN2 was validated using ELISA with an increasing number of plasma samples (PsP and recurrence groups, n = 8 and n = 37, respectively). A significant difference in serum ATXN2 levels was observed between the PsP and recurrence groups (mean [ng/mL] ± standard deviation [SD], 8.5 ± 2.6, 15.1 ± 1.2, respectively; p = 0.028) (Fig. 2a). We then calculated standardized residuals using months after chemoradiotherapy as a covariate because there was a significant group difference for this variable. The results showed that the difference between groups remained significant (p = 0.045, Supplementary Fig. 3a). When the same analysis was performed in the proteomics cohort (N = 14) and the cohort excluding proteomics analysis (N = 31), a similar trend was observed in the former; however, the observed trend did not reach significance given the small sample size (p = 0.16, Supplementary Fig. 3b). In contrast, the difference remained significant in the non-proteomics cohort (p = 0.040, Supplementary Fig. 3c). No significant differences were noted based on demographic and clinical factors, such as age, sex, the extent of resection, methylguanine-DNA methyltransferase promoter methylation, laterality, and the location of the enhanced lesion between the groups (Supplementary Table 5). However, the sample collection time (months after chemoradiotherapy) was significantly longer in the recurrence group than in the PsP group (mean ± SD, 10.5 ± 11.5 months and 4.1 ± 3.2, respectively; p = 0.038).

We analyzed the cut-off point to distinguish the disease status using ATXN2 as an explanatory variable via ROC analysis. The specificity for differentiating recurrence from PsP was high (100%), indicating the potential for independent clinical utility. However, the sensitivity was insufficient (59.5%, Fig. 2b). The cut-off value was 12.8 ng/mL, which was set as the concentration threshold. The recurrence group was divided into low- and high-ATXN2-expression subgroups (n = 20 and n = 17, respectively) based on this threshold. The mean ATXN2 concentrations in the high- and low-expression groups were 20.7 ng/mL and 8.7 ng/mL, respectively (Fig. 2a). We then considered two factors—ATXN2 expression and months after chemoradiotherapy—as explanatory variables, since the duration after treatment is an essential factor in estimating disease progression in clinical practice. ATXN2 levels of ≥ 11.0 ng/mL and a duration of ≥ 8 months after chemoradiotherapy were associated with a high probability of predicting recurrence (area under the curve = 0.82, sensitivity = 67.6%, and specificity = 87.5) (Fig. 2c). To confirm the results, we conducted the same analysis using only one factor—months after chemoradiotherapy—but found lower accuracy in distinguishing recurrence from PsP (AUC = 0.73, sensitivity = 54.1%, specificity = 87.5) (Supplementary Fig. 4a). We then compared the AUC values of three models using different explanatory variables: ATXN2 alone, months after chemoradiotherapy alone, and ATXN2 and months after chemoradiotherapy combined. Although a significant difference was observed among the models (p = 0.0001), the pairwise analysis revealed that the combined model significantly outperformed the ATXN2-only model (p = 0.0079) but did not significantly outperform the months-only model (p = 0.17). However, the AUC of the combined model was higher, and its 95% confidence interval (0.64–0.92) shifted upward compared to that of the months-only model (0.52–0.88). This result suggests a potential trend toward improved discriminatory performance (Supplementary Fig. 4b).

We analyzed the ATXN2 expression in three and four tissue samples from the low- and high-expression groups, respectively, using western blotting (Fig. 2d). Despite one outlier, ATXN2 levels were generally lower in the low-expression samples, which was consistent with the ELISA results. However, the small sample size did not permit statistically meaningful group comparisons.

### Expression of ATXN2 in glioma tissues

**Fig. 3:**
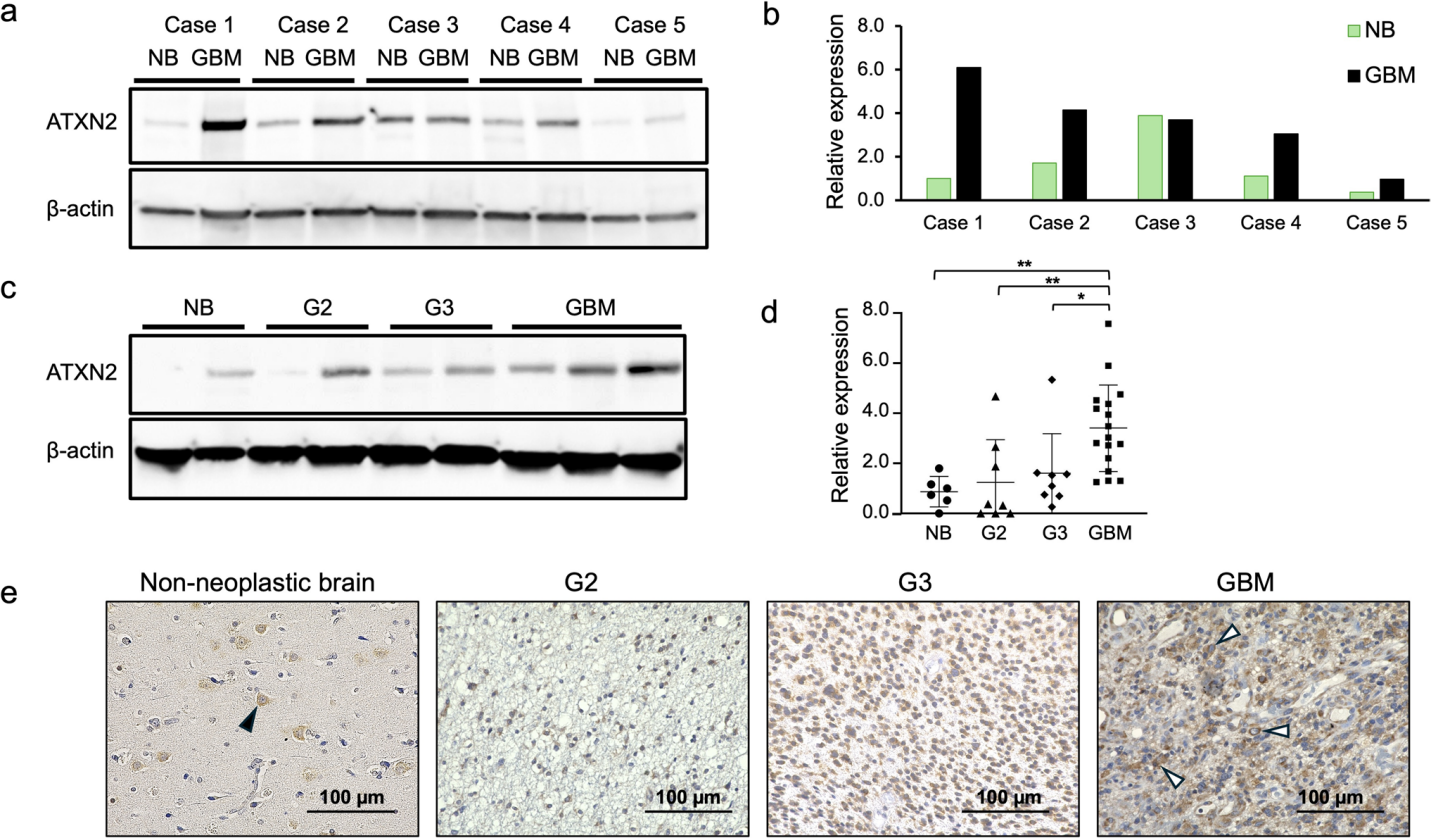
Western blotting and immunohistochemistry of ATXN2 in tissue samples. **a.** ATXN2 expression was higher in GBM tissue samples compared to that in lower-grade gliomas and non-tumor tissue. ATXN2 levels were examined in five pairs of normal brain (NB) tissues and GBM tissues using western blotting. **b.** In four of the five samples, ATXN2 expression, normalized to β-actin, was higher in GBM samples compared to those in non-tumor tissues. Green bar, normal brain; black bar, GBM brain. **c.** Western blot analysis for ATXN2 expression in non-tumor brain tissue (NB) and various grades of glioma. **d.** ATXN2 levels in normal brain, glioma grade 2, grade 3, and GBM tissues. **e.** Immunohistochemistry assessed using normal brain, glioma grade 2, grade 3, and GBM slides. Neurons (black triangles) in NB tissues and GBM cells (white triangles) expressed ATXN2. Paraffin sections were immunostained with antibodies against ATXN2, with a hematoxylin counterstain. Original magnification × 400. NB, normal brain; GBM, glioblastoma; G2, grade 2; G3, grade 3. *p < 0.05; **p < 0.01

To evaluate ATXN2 expression in tissues, western blotting was conducted using five GBM samples, including both tumor and adjacent non-tumor brain tissues (Fig. 3a). Western blot band densities were standardized with β-actin controls. ATXN2 expression was higher in tumor tissues than in normal brain tissues in four of the five samples (Fig. 3b). We then compared ATXN2 expression based on different tumor grades, including normal brain (n = 6), grade 2 (n = 8), grade 3 (n = 8), and GBM (n = 17). ATXN2 expression was higher in GBM tissues than in lower-grade gliomas or non-neoplastic brain tissues (Fig. 3c, Supplementary Fig. 5). Statistical analyses revealed that ATXN2 was significantly more highly expressed in GBM than in non-neoplastic tumor, grade 2, and grade 3 glioma groups (p = 0.0054, p = 0.0088, and p = 0.035, respectively) (Fig. 3d).

Immunohistochemistry using an ATXN2 antibody revealed that ATXN2 was expressed in the cytoplasm of neurons and tumor cells (Fig. 3e). To evaluate the expression pattern of ATXN2 in GBM tissue, additional immunohistochemical and H&E staining was performed on sections of GBM and normal brain samples. Histological slides were prepared and independently reviewed by an experienced neuropathologist (HS). In the normal brain tissue, ATXN2 staining was absent in vascular endothelial cells (Supplementary Fig. 6a). In contrast, endothelial cells lining the blood vessels within the tumor microenvironment were faintly stained (Supplementary Fig. 6b). Furthermore, ATXN2 expression was high in tumor cells (Supplementary Fig. 6c). No obvious staining was observed in immune cells represented by macrophages in GBM tissue.

Our findings therefore indicate that ATXN2 expression is predominantly localized to tumor cells. Although weak ATXN2 staining was observed in some endothelial cells within the tumor microenvironment, the signal intensity was considerably lower than that seen in malignant cells. Thus, tumor cells constitute the main source of ATXN2.

### *ATXN2* knockdown in glioma cell lines

**Fig. 4:**
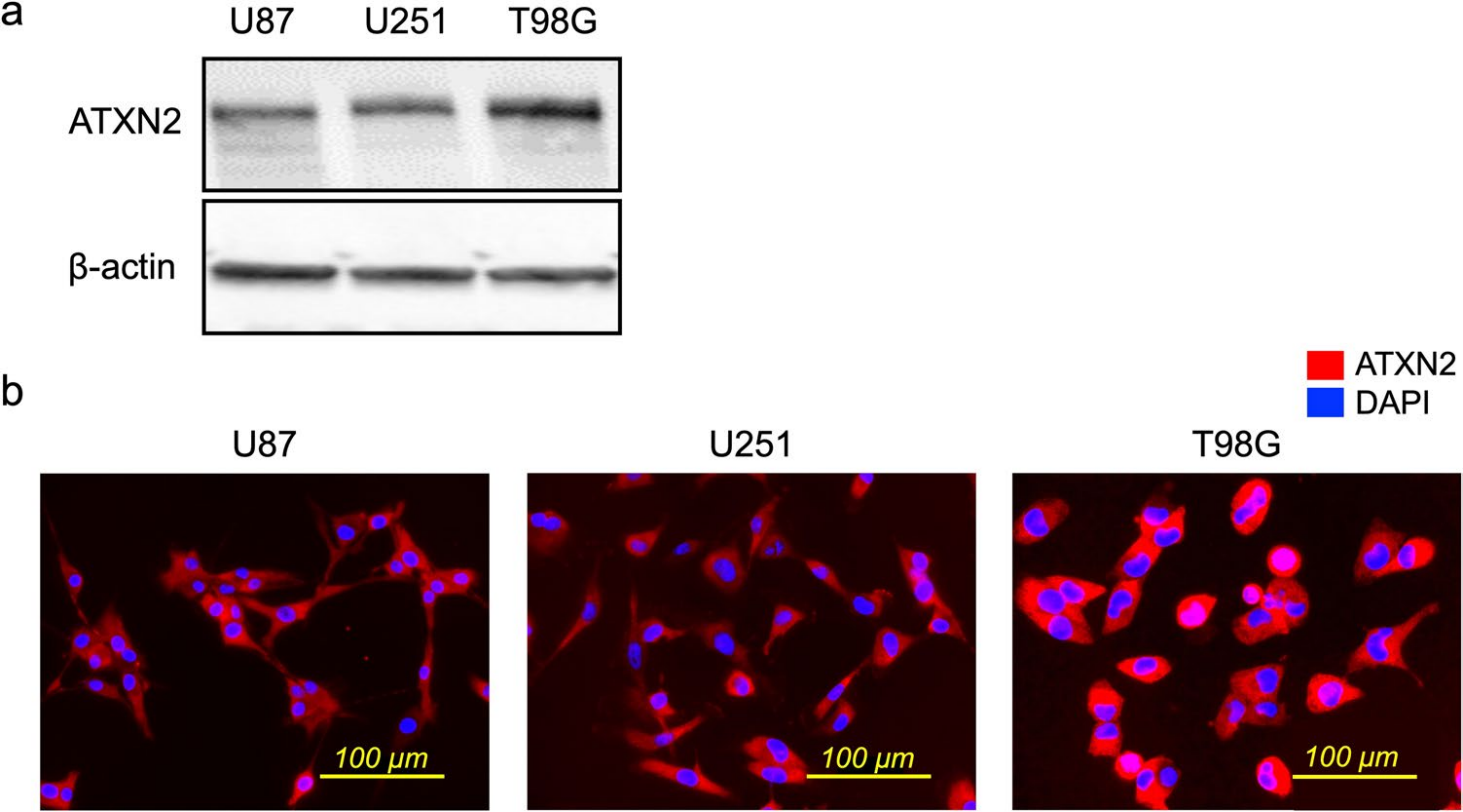
ATXN2 expression in glioma cell lines. **a.** ATXN2 expression in glioma cell lines (U87, U251, and T98G) was determined using western blotting. **b.** Immunofluorescent staining showing ATXN2 (red) localized in the cytoplasm. Blue, DAPI. Red, ATXN2; blue, DAPI

Western blotting and immunocytochemistry analyses were performed to further explore ATXN2 and its expression in glioma cell lines. Western blot analysis of proteins revealed ATXN2 expression in all (U87, U251, and T98G) cell lines (Fig. 4a). Furthermore, cytoplasmic localization of ATXN2 was observed in all three cell lines (Fig. 4b), with the highest intensity in the T98G cell line, consistent with the western blot data.

**Fig. 5:**
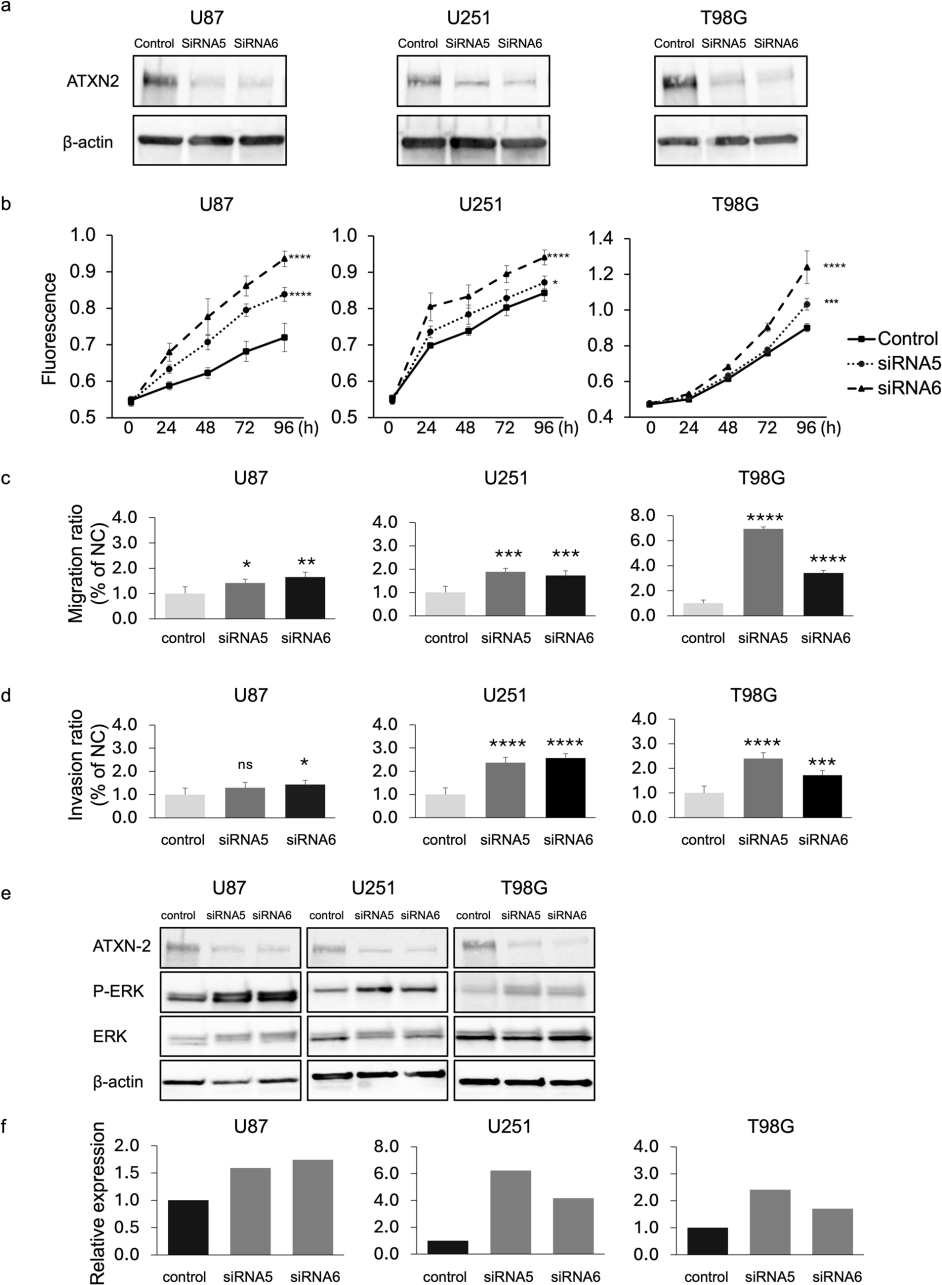
Effect of *ATXN2* knockdown in glioma cell lines. **a.** *ATXN2* knockdown enhanced glioma cell proliferation, migration, and invasion and was associated with increased p-ERK expression. The successful knockdown of *ATXN2* was confirmed using western blot analysis, demonstrating a reduction in ATXN2 protein levels in the glioma cell lines treated with siATXN2-5 and siATXN2-6 compared to those in the negative control. **b.** Proliferation assay performed using Alamar blue stain. Both types of ATXN2 siRNAs decreased cell viability. **c.** Migration ability evaluated using the Transwell assay. Transfected cells were stained and counted to determine the invasion ability. **d**. Invasion ability evaluated using the Transwell assay. **e.** Following *ATXN2* knockdown, glioma cells exhibited increased p-ERK expression. **f.** p-ERK levels in glioma cells following *ATXN2* knockdown were quantified and standardized based on β-actin levels. NC, negative control. Bars represent mean values ± SD. *p < 0.05; **p < 0.01; ***p < 0.001; ****p < 0.0001

Functional assays were performed to elucidate the role of ATXN2 in glioma cells. *ATXN2* knockdown using siRNA efficiently reduced its expression at both mRNA and protein levels (Fig. 5a and Supplementary Fig. 7). Cell proliferation increased in siATXN2-5- and siATXN2-6-treated cells compared with control: U87 (16.4 and 30.0%, p < 0.0001 and p < 0.0001, respectively), U251 (3.4% and 11.6%, p = 0.017 and p < 0.0001, respectively), and T98G (14.6 and 37.7%, p = 0.0004 and p < 0.0001, respectively) (Fig. 5b). Furthermore, the Transwell migration assay revealed that groups treated with siATXN2-5 and siATXN2-6 exhibited higher migratory abilities than the negative control group: U87 (1.4- and 1.6-fold increase; p = 0.0168 and p = 0.0007, respectively), U251 (1.9 and 1.7-fold increase; p < 0.0001 and p < 0.0001, respectively), and T98G (6.9 and 3.4-fold increase, p < 0.0001 and p < 0.0001, respectively) (Fig. 5c). Similarly, *ATXN2* knockdown led to increased invasiveness in U87 (1.3- and 1.4-fold; p = 0.13 and p = 0.026, respectively), U251 (2.4- and 2.6-fold, p < 0.0001 and p < 0.0001, respectively), and T98G (2.4- and 1.7-fold; p < 0.0001 and p = 0.002, respectively) cell lines than that in the negative control (Fig. 5d). These results suggest that ATXN2 expression was negatively correlated with the proliferative and invasive ability of glioma cells.

We investigated the expression of proteins downstream of the ATXN2 signaling pathway. The results showed that p-ERK expression was increased after *ATXN2* knockdown in all cell lines used as follows: U87 cells showed a 1.8- and 1.9-fold increase, U251 cells showed approximately 3.8 and 3.1-fold increase, and T98G cells showed approximately 2.4- and 1.9-fold increase (Fig. 5e and f). These results suggest that ATXN2 may regulate glioma cell proliferation via the ERK signaling pathway. No changes were observed in Akt, mammalian target of rapamycin, or signal transducer and activator of transcription 3 phosphorylation (Supplementary Fig. 8).

## Discussion

This study indicated that plasma ATXN2 may be able to discriminate between GBM recurrence and PsP defined via SWATH MS and ELISA. Both in vivo and in vitro experiments showed that ATXN2 was highly expressed in GBM cells. Knockdown of ATXN2 promoted cell proliferation, migration, and invasion activity in GBM cells. Accompanying these phenotypic changes, ERK was phosphorylated.

Plasma ATXN2 levels reflect its expression in GBM tissues because the high-plasma-ATXN2 group exhibited high expression in tumor tissues. In GBM tissues, the main resource of ATXN2 is tumor cells. These results suggest a potential role for ATXN2 as a blood biomarker for detecting recurrence. However, ATXN2 alone cannot completely distinguish PsP from recurrence because ATXN2 expression varied in GBM. In this context, the combination of ATXN2 and months after chemoradiotherapy (i.e., ≥ 8 months) are reasonable factors because duration after treatment is a key factor for estimating disease status in clinical practice.

Previous studies have reported the function of ATXN2 in tumors; however, the effect of ATXN2 may differ across various tumor types. In esophageal squamous cell carcinoma, ATXN2 is associated with poor prognosis [24]. ATXN2 also facilitates chemoresistance to 5-fluorouracil chemotherapy in gastric cancer [25]. In contrast, high ATXN2 protein levels promote apoptosis in neuroblastoma cells [26], suggesting its protective role against tumors, in agreement with our results. However, to our knowledge, the involvement of ATXN2 in GBM has not been previously reported, and its potential role in glioma biology remains largely unexplored. Investigating this association was a one of the central aims of our study and represents a key novel contribution to the literature.

Western blotting analysis in glioma cells treated with siATXN2s showed phosphorylation of ERK, which is one of the most important signaling pathways in GBM. ERK1/2 promotes glioma cell proliferation, migration, and invasion and leads to poor prognosis in patients with glioma [27–29]. Consistent with the aforementioned studies, our results showed high proliferation activity in glioma cells following ATXN2 inhibition. Unexpectedly, our results showed that serum ATXN2 levels increased in tumor relapse cases, whereas biological experiments revealed that ATXN2 expressed from tumors suppressed the malignant phenotype of GBM cells. As previously shown in other cancers and GBM [30, 31], biomarkers produced by tumor cells do not always promote malignancy. Recent findings also suggest that RNA-binding proteins involved in alternative splicing can exert either oncogenic or tumor-suppressive effects depending on the cellular context, tumor stage, and environmental stressors, supporting the hypothesis that ATXN2 may play a similarly context-dependent role in glioma [32].

The elevated expression of ATXN2 in recurrent gliomas may represent a compensatory tumor-suppressive response to oncogenic stress. Such adaptive induction of tumor suppressors is a mechanism that limits malignant progression. For example, p16^INK4a^ is upregulated by oncogenic signaling pathways (e.g., BRAF^V600E^), serving as a transient barrier to tumor growth despite its low basal levels in normal cells [33]. By analogy, the increased expression of ATXN2 in GBM may represent a similar attempt to counteract oncogenic signals. Further studies will be required to determine whether this response exerts functional tumor-suppressive effects or instead marks persistent cellular stress in the tumor microenvironment.

The current study had some limitations. Strict classification of samples as PsP or recurrence is challenging because both conditions can coexist in different regions of the same lesion, resulting in a heterogeneous pathology [34]. We cannot exclude the possibility that some of the patients with PsP may have recurrent tumor tissue. The relationship between imaging methods and plasma ATXN2 expression level remains unclear, particularly regarding the threshold of changes in the images that correspond to those in ATXN2 levels. Regarding the literature review, there are potential concerns with publication availability and selection bias. Another limitation of this study is the lack of overexpression experiments to complement the knockdown data. Although our findings suggest a tumor-suppressive role for ATXN2, further studies are needed to confirm its function and underlying mechanisms. The purpose of this study was to distinguish PsP from true recurrence of GBM. Therefore, we did not include healthy control subjects, as differentiating between tumor and non-tumor tissues was beyond the scope of the study. However, extending our analyses to include differentiation from non-tumor tissue is a key direction for future research. Most importantly, the size of tissue and serum samples, particularly for PsP samples, was insufficient. Future studies with larger cohorts are required to validate the results of this study and assess their clinical significance.

In conclusion, ATXN2 shows potential as a blood biomarker for estimating disease status when GBM recurrence is suspected.

## Supplementary Information

Below is the link to the electronic supplementary material.

Supplementary file1 (JPG 112 KB)

Supplementary file2 (JPG 226 KB)

Supplementary file3 (JPG 642 KB)

Supplementary file4 (JPG 675 KB)

Supplementary file5 (JPG 332 KB)

Supplementary file6 (JPG 2345 KB)

Supplementary file7 (JPG 230 KB)

Supplementary file8 (JPG 699 KB)

Supplementary file9 (XLSX 15 KB)

Supplementary file10 (XLSX 2325 KB)

Supplementary file11 (XLSX 33 KB)

Supplementary file12 (DOCX 27 KB)

Supplementary file13 (DOCX 21 KB)

## Data Availability

Not applicable.
