# Supplementary figures and images for: Ataxin-2 as a candidate blood biomarker for estimating disease status in cases of suspected glioblastoma recurrence
Source: Brain Tumor Pathol. 2025 Sep 22;43(2):43–55. doi: 10.1007/s10014-025-00517-z (PMC13076406; doi:10.1007/s10014-025-00517-z)

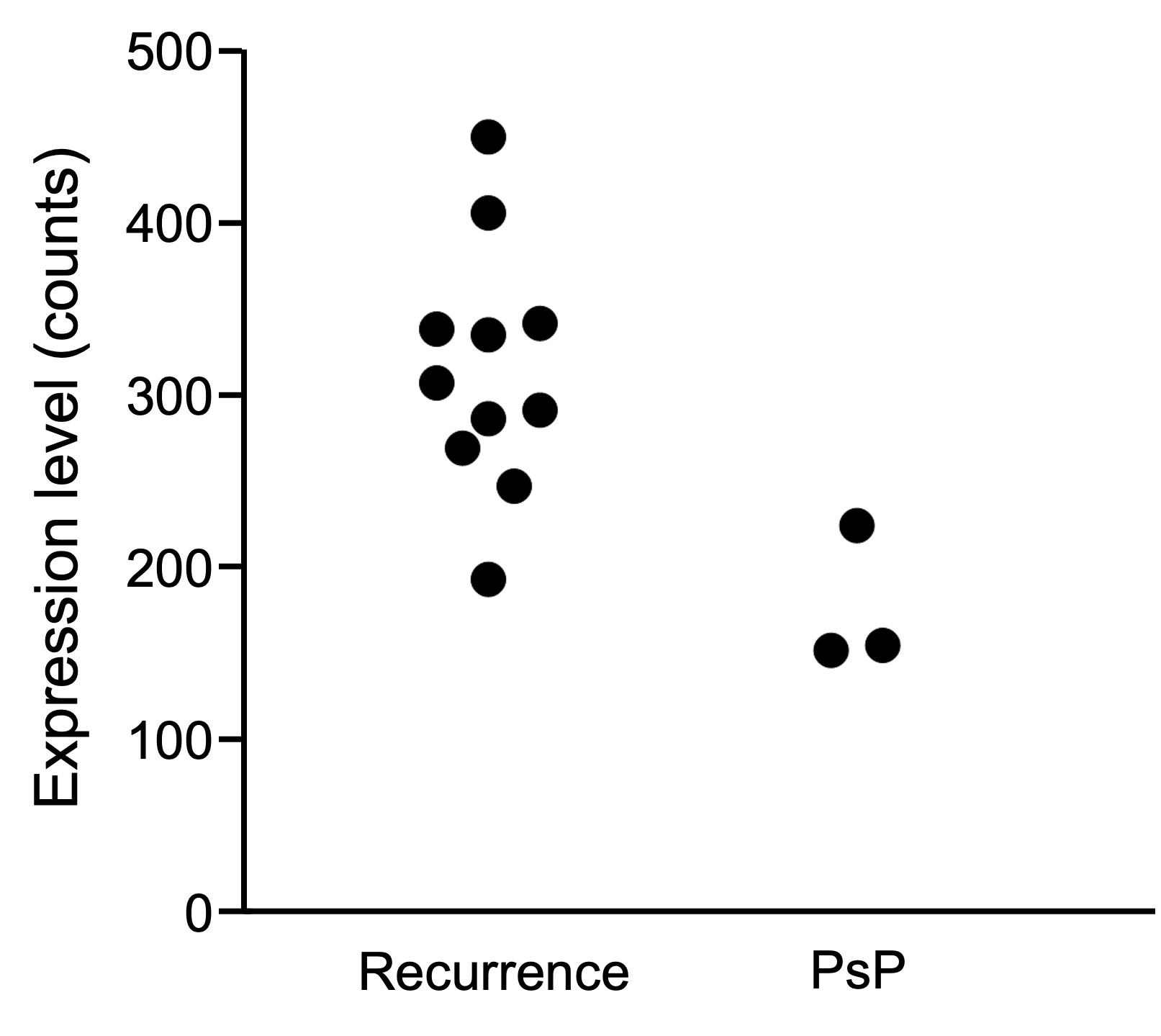

Supplement: Supplementary file 1 — Supplementary file1 (JPG 112 KB) [file 10014_2025_517_MOESM1_ESM.jpg]

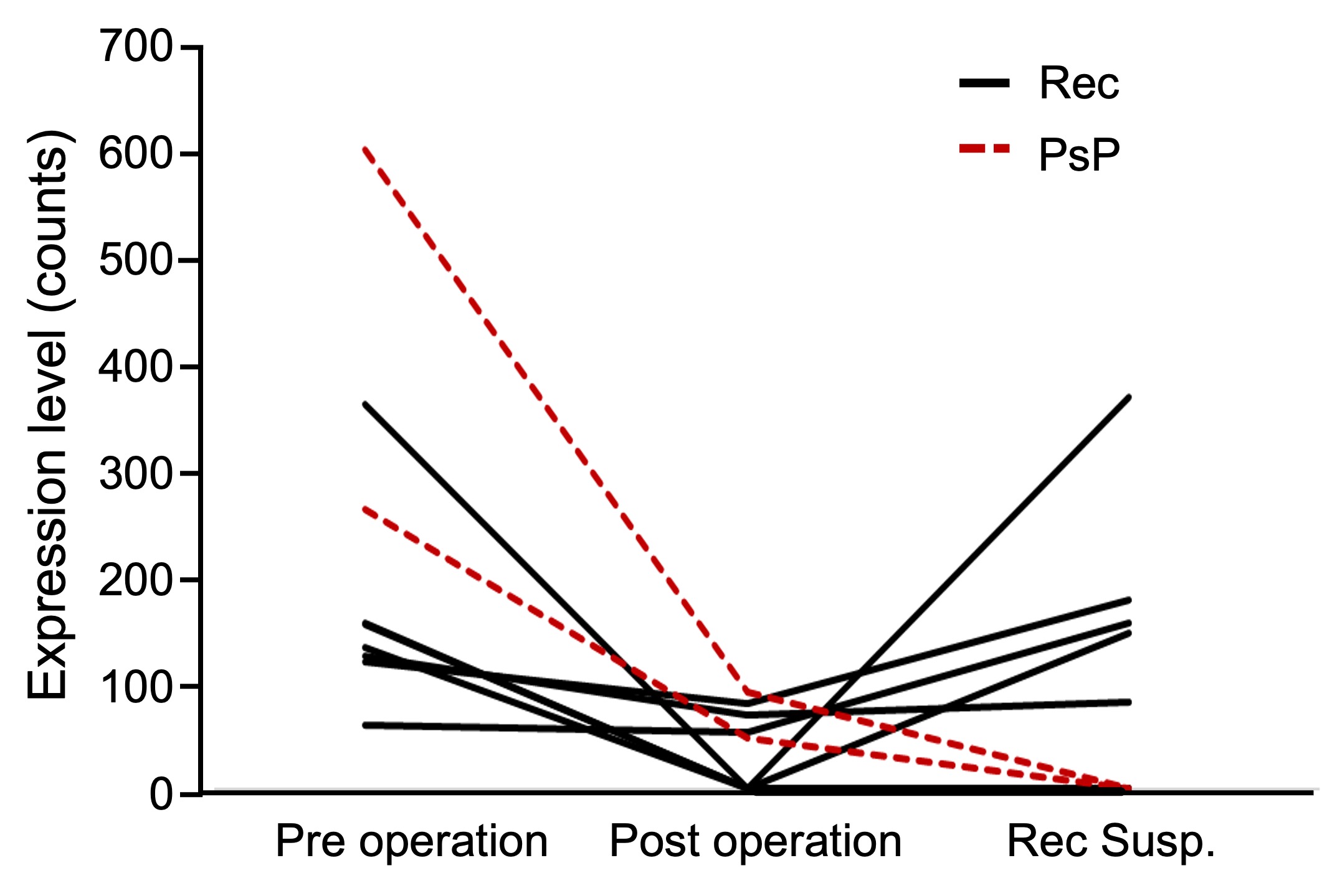

Supplement: Supplementary file 2 — Supplementary file2 (JPG 226 KB) [file 10014_2025_517_MOESM2_ESM.jpg]

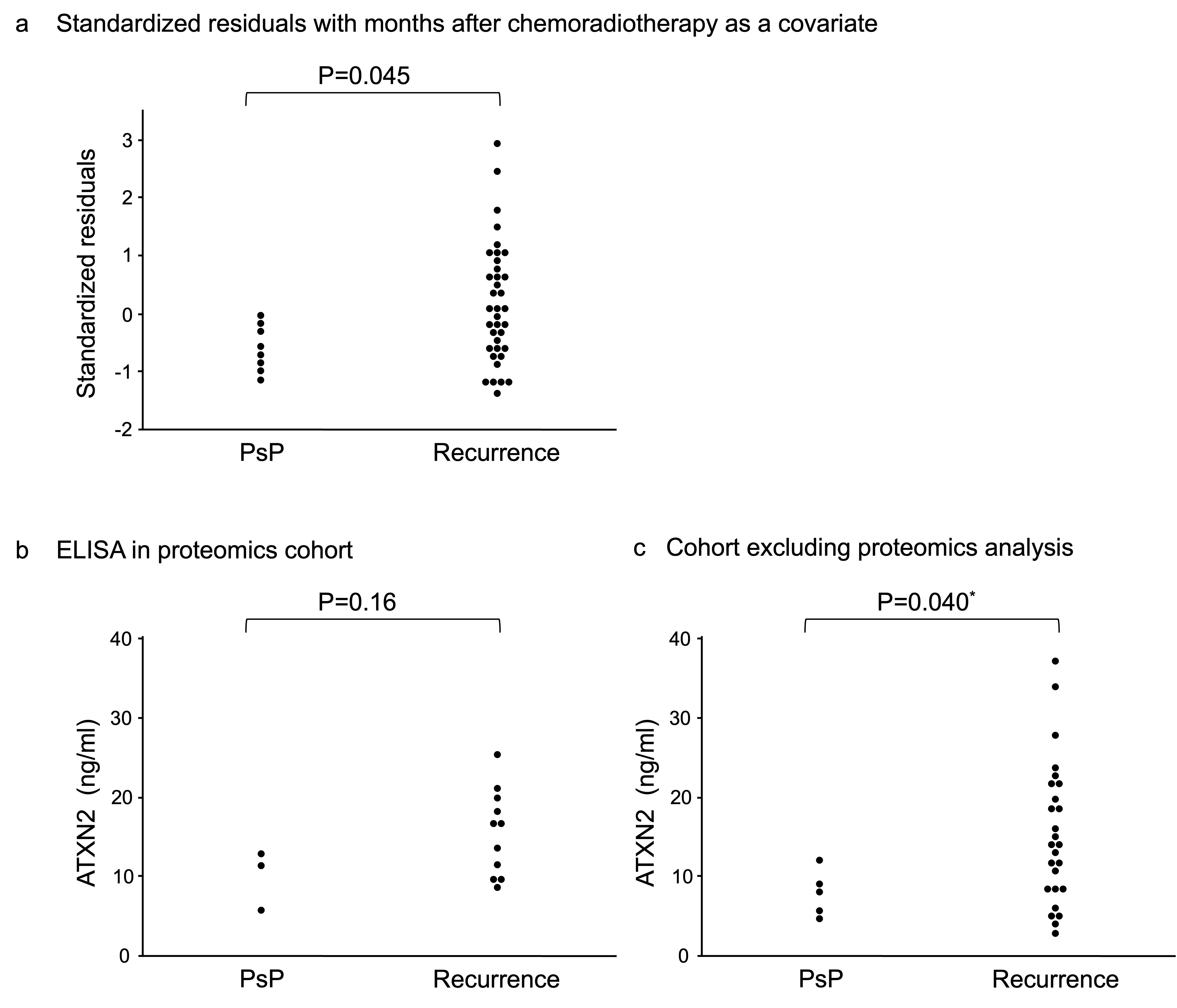

Supplement: Supplementary file 3 — Supplementary file3 (JPG 642 KB) [file 10014_2025_517_MOESM3_ESM.jpg]

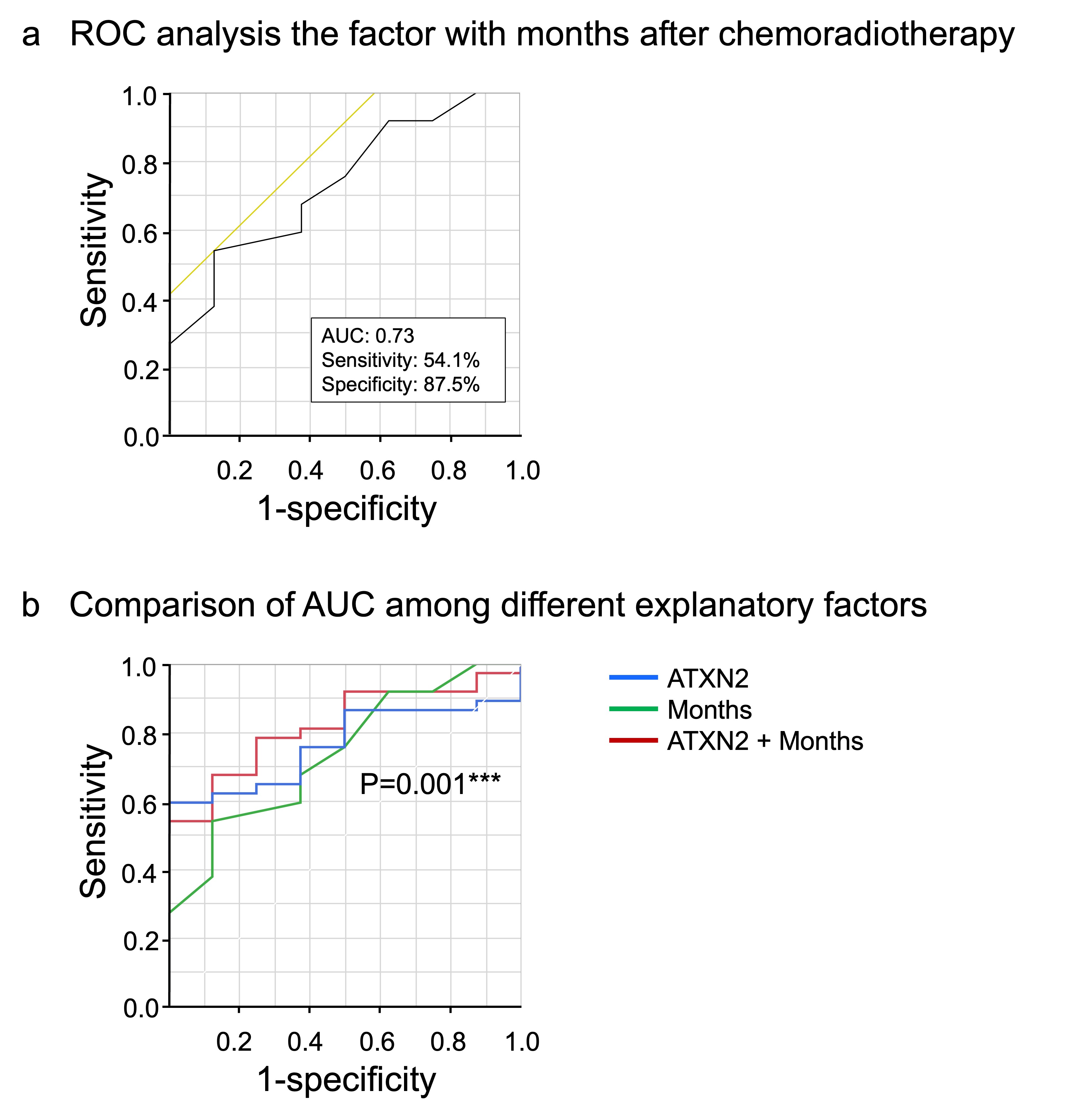

Supplement: Supplementary file 4 — Supplementary file4 (JPG 675 KB) [file 10014_2025_517_MOESM4_ESM.jpg]

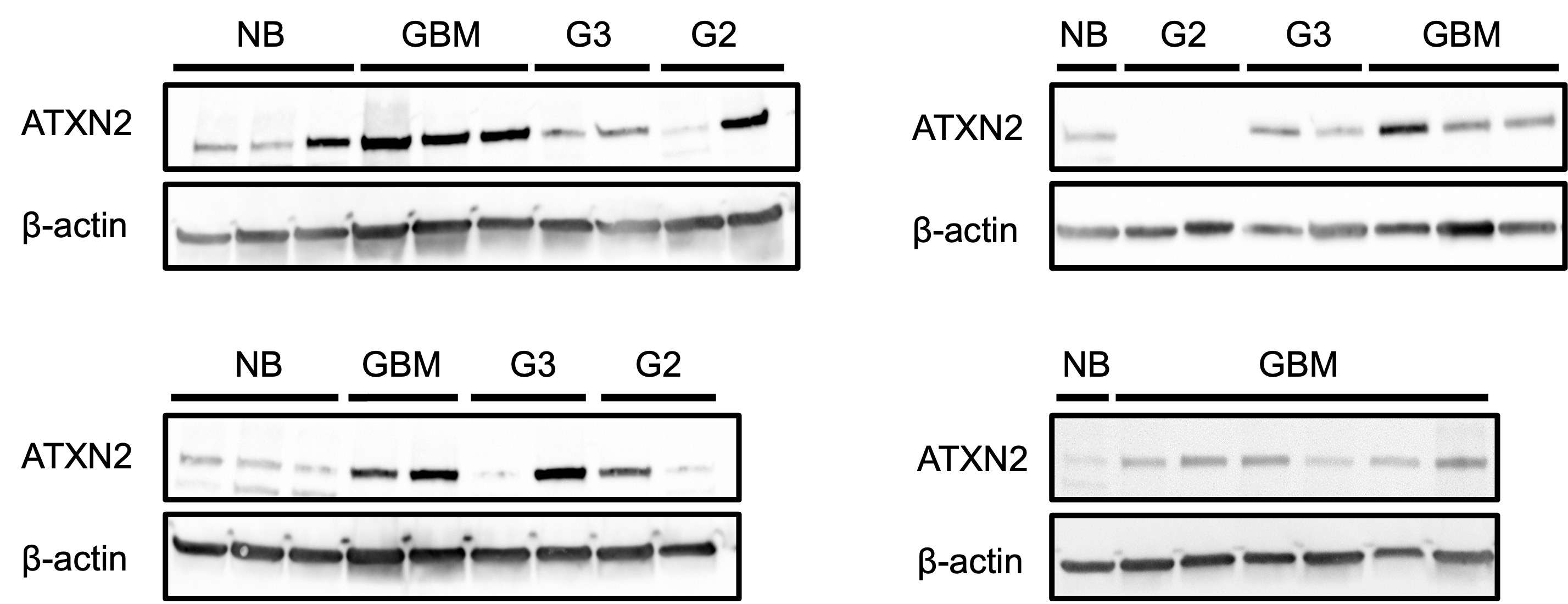

Supplement: Supplementary file 5 — Supplementary file5 (JPG 332 KB) [file 10014_2025_517_MOESM5_ESM.jpg]

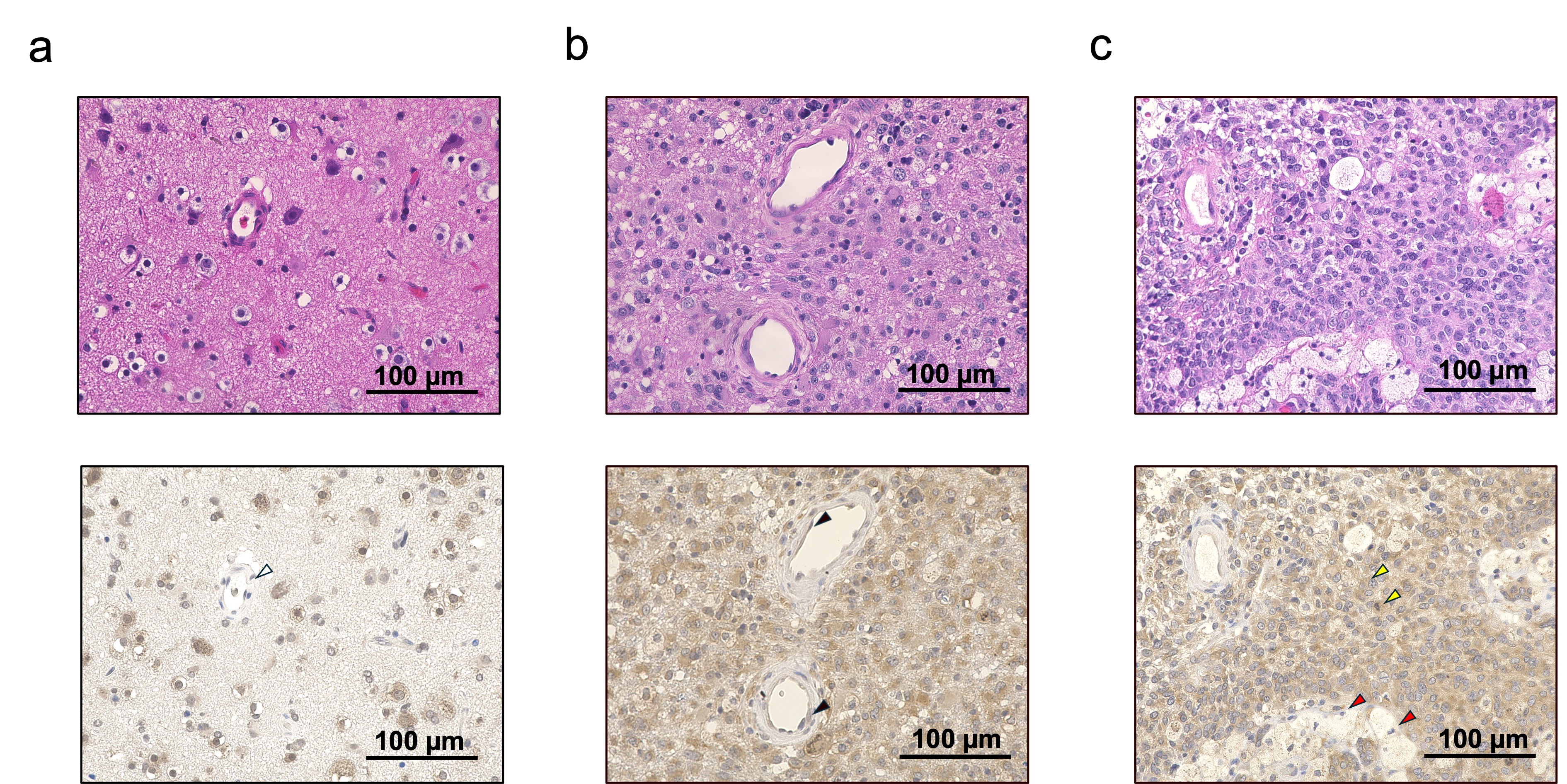

Supplement: Supplementary file 6 — Supplementary file6 (JPG 2345 KB) [file 10014_2025_517_MOESM6_ESM.jpg]

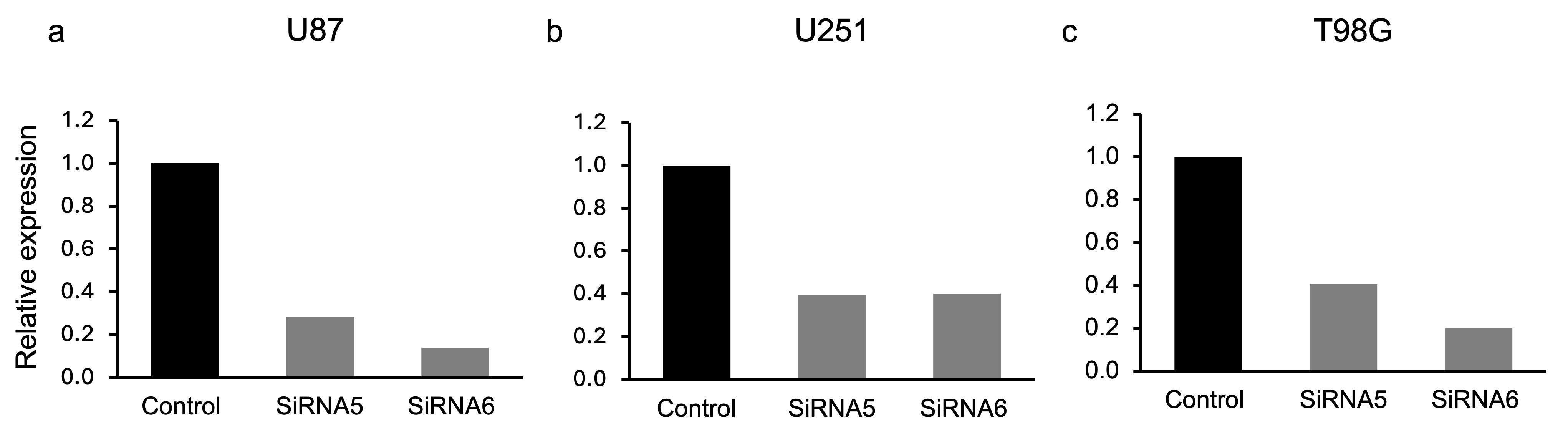

Supplement: Supplementary file 7 — Supplementary file7 (JPG 230 KB) [file 10014_2025_517_MOESM7_ESM.jpg]

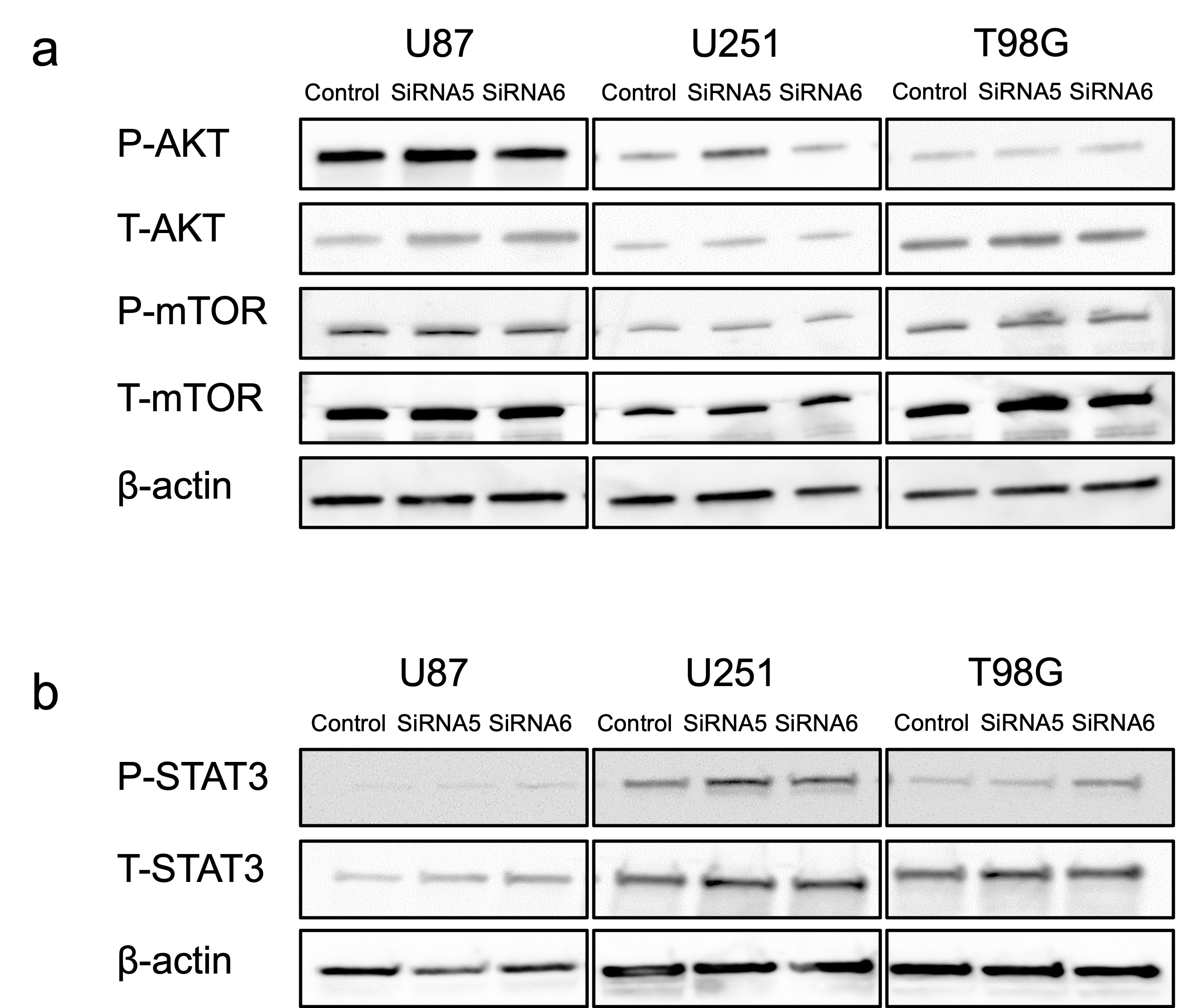

Supplement: Supplementary file 8 — Supplementary file8 (JPG 699 KB) [file 10014_2025_517_MOESM8_ESM.jpg]
