# Supplementary material for: Ataxin-2 as a candidate blood biomarker for estimating disease status in cases of suspected glioblastoma recurrence
Source: Brain Tumor Pathol. 2025 Sep 22;43(2):43–55. doi: 10.1007/s10014-025-00517-z (PMC13076406; doi:10.1007/s10014-025-00517-z)
Supplement: Supplementary file 12 — Supplementary file12 (DOCX 27 KB) [file 10014_2025_517_MOESM12_ESM.docx]

**Supplementary Table 1.** Demographic and clinical characteristic of participants for proteomics analysis (N=14)

| **Characteristics** | **True recurrence**  **(n=11)** | **Pseudo-progression**  **(n=3)** |
| --- | --- | --- |
| Age |  |  |
| Mean ± SD | 70.2 ± 8.1 | 61.0 ± 10.8 |
| Range | 56 to 83 | 52 to 73 |
| Sex |  |  |
| Male | 6 (55%) | 1 (33%) |
| Female | 5 (45%) | 2 (67%) |
| Surgery |  |  |
| Surgery for resection | 11 (100%) | 3 (100%) |
| Biopsy | 0 (0%) | 0 (0%) |
| Extent of resection (%) | 99.6 ± 0.8 | 99.6 ± 0.5 |
| IDH mutation |  |  |
| Wild type | 11 | 3 |
| Mutant | 0 | 0 |
| MGMT promoter methylation |  |  |
| Methylated | 5 (45%) | 1 (33%) |
| Unmethylated | 6 (55%) | 2 (67%) |
| Months after the chemoradiotherapy |  |  |
| Mean ± SD | 18.1 ± 16.6 | 6.0 ± 3.6 |
| Range | 4 to 58 | 2 to 9 |
| Laterality of enhanced lesion |  |  |
| Left | 8 (73%) | 1 (33%) |
| Right | 3 (27%) | 2 (67%) |
| Location of enhanced lesion |  |  |
| Frontal | 3 | 2 |
| Parietal | 1 | 0 |
| Temporal | 4 | 0 |
| Occipital | 1 | 1 |
| Insula | 2 | 0 |

IDH, Isocitrate dehydrogenase; O^6^-methylguanine-DNA methyltransferase, MGMT.

**Supplementary Table 5**. Demographic and clinical characteristic of participants

| **Characteristics** | **True recurrence**  **(n=37)** | **Pseudo-progression**  **(n=8)** | **P-value** |
| --- | --- | --- | --- |
| Age |  |  | 0.64 |
| Mean ± SD | 60.9 ± 12.1 | 62.5 ± 16.2 |  |
| Range | 39 to 83 | 32 to 93 |  |
| Sex |  |  | 0.11 |
| Male | 23 (62%) | 2 (25%) |  |
| Female | 14 (38%) | 6 (75%) |  |
| Surgery |  |  | 0.18 |
| Surgery for resection | 37 (100%) | 7 (88%) |  |
| Biopsy | 0 (0%) | 1 (12%) |  |
| Extent of resection (%) | 93.1 ± 16.2 | 98.1 ± 2.0 | 0.30 |
| IDH mutation |  |  | 1.0 |
| Wild type | 37 (100%) | 8 (100%) |  |
| Mutant | 0 (0%) | 0 (0%) |  |
| MGMT promoter methylation |  |  | 1.0 |
| Methylated | 16 (43%) | 3 (38%) |  |
| Unmethylated | 21 (57%) | 5 (62%) |  |
| Months after the chemoradiotherapy |  |  | 0.038^*^ |
| Mean ± SD | 10.5 ± 11.5 | 4.1 ± 3.2 |  |
| Range | 1 to 58 | 0 to 9 |  |
| Laterality of enhanced lesion |  |  | 1.0 |
| Left | 20 (54%) | 4 (50%) |  |
| Right | 17 (46%) | 4 (50%) |  |
| Location of enhanced lesion |  |  | 0.15 |
| Frontal | 12 | 6 |  |
| Parietal | 5 | 0 |  |
| Temporal | 15 | 1 |  |
| Occipital | 2 | 1 |  |
| Insula | 3 | 0 |  |

IDH, Isocitrate dehydrogenase; O^6^-methylguanine-DNA methyltransferase, MGMT. Fisher’s exact test or Mann-Whitney *U* test were performed. ^*^p < .05
