# Supplementary material for: Ataxin-2 as a candidate blood biomarker for estimating disease status in cases of suspected glioblastoma recurrence
Source: Brain Tumor Pathol. 2025 Sep 22;43(2):43–55. doi: 10.1007/s10014-025-00517-z (PMC13076406; doi:10.1007/s10014-025-00517-z)
Supplement: Supplementary file 13 — Supplementary file13 (DOCX 21 KB) [file 10014_2025_517_MOESM13_ESM.docx]

*Brain Tumor Pathology*

Supplementary material for: **Ataxin-2 as a candidate blood biomarker for estimating disease status in cases of suspected glioblastoma recurrence**

Farida Garaeva, MD, Riho Nakajima, OT, PhD, Sho Tamai, MD, PhD, Kensuke Tateishi, MD, PhD, Akitake Mukasa, MD, PhD, Shinji Kawabata, MD, PhD, Hiroaki Nagashima, MD, PhD, Manabu Natsumeda, MD, PhD, Nozomi Hirai, MD, Shota Tanaka, MD, PhD, Shigeo Ohba, MD, PhD, Nayuta Higa, MD, PhD, Yoshiki Arakawa, MD, PhD, Akihide Kondo, PhD, MD, Hidehiro Kohzuki, PhD, MD, Shinichiro Koizumi, PhD, MD, Yutaka Fujioka, PhD, MD, Tatsuya Abe, PhD, MD, Hemragul Sabit, PhD, Masashi Kinoshita, PhD, MD, Yasuo Uchida, PhD, Sumio Ohtsuki, PhD, Mitsutoshi Nakada, MD, PhD*****

*** Corresponding author:** Mitsutoshi Nakada,

Department of Neurosurgery, Graduate School of Medical Science, Kanazawa University, 13-1 Takara-machi, Kanazawa 920-8641, Japan.

Phone: +81-76-265-2383, FAX: +81-76-234-4262

Email: [mnakada@med.kanazawa-u.ac.jp](mailto:mnakada@med.kanazawa-u.ac.jp)

**Supplementary Figure 1.** Protein X was highly expressed in serum samples of patients with glioblastoma recurrence (N = 11) but was expressed at a significantly lower level in patients with pseudoprogression (PsP) (N = 3) (p = 0.0052).

**Supplementary Figure 2.** Dynamics of serum ATXN2 levels in glioblastoma progression. Serum ATXN2 levels were higher before surgical treatment, decreased postoperatively, and increased after glioblastoma recurrence. Susp, suspected; Rec, recurrence; PsP, pseudoprogression.

**Supplementary Figure 3a**. Standardized residuals, adjusted for months after chemoradiotherapy, showed a significant group difference (p = 0.045). **b.** ATXN2 expression in plasma, measured using ELISA, was evaluated in the proteomics cohort (N = 14). Although ATXN2 levels remained higher in patients with recurrence than in those with pseudoprogression (PsP), the difference was not significant (p = 0.14). **c.** ATXN2 expression, measured using ELISA, was evaluated in the cohort excluding proteomics analysis (N = 31). ^*^p < 0.05. **c.** Receiver operating characteristic (ROC) curve analyses were performed using months after chemoradiotherapy as an explanatory variable.

**Supplementary Figure 4a.** Receiver operating characteristic (ROC) analyses were performed using months after chemoradiotherapy (Months) as an explanatory variable. **b.** AUC was compared among different explanatory factors, including ATXN2, Months, and ATXN2 plus Months after chemoradiotherapy. ^***^p < 0.001

**Supplementary Figure 5.** ATXN2 expression in tissue samples. ATXN2 expression is increased in glioblastoma (GBM) tissue. ATXN2 expression in tissue samples of normal brain (NB), glioma grades 2 and 3, and GBM examined by western blotting. The NB in the leftmost column serves as a control sample, which appears second from the left in Figure 3C.

**Supplementary Figure 6.** Representative images of glioblastoma (GBM) and normal brain tissues stained with hematoxylin and eosin and immunohistochemistry for ATXN2. a. Immunohistochemistry shows no detectable ATXN2 staining in normal brain blood vessels (white arrow). b. In contrast, vascular endothelial cells in GBM tissue (black arrow) exhibit faint ATXN2 staining. c. Tumor cells (yellow arrow) display strong ATXN2 positivity, whereas immune cells (macrophages) (red arrow) demonstrated no obvious staining.

**Supplementary Figure 7.** mRNA levels of ATXN2 in glioma cell lines. *ATXN2* mRNA level after knockdown. ATXN2 mRNA expression is significantly reduced following knockdown in glioma cell lines, U87 (**a**), U251 (**b**), and T98G (**c**).

**Supplementary Figure 8.** Alteration of various signaling pathways in cell lines transfected with siRNAs. Western blot analysis of total AKT, p-AKT, total mTOR, p-mTOR, total STAT, and p-STAT3 (Y705) in cells transfected with siATXN2s or negative control siRNA.
